# Development and Validation of an HPLC–UV/PDA Method for the Determination of Cannflavins in Different *Cannabis sativa* Chemovars

**DOI:** 10.3390/mps8050100

**Published:** 2025-09-03

**Authors:** Mostafa A. Elhendawy, Mohamed M. Radwan, Elsayed A. Ibrahim, Amira S. Wanas, Adel A. Marzouk, Suman Chandra, Murelle Godfrey, Mahmoud A. ElSohly

**Affiliations:** 1 National Center for Natural Products Research, School of Pharmacy, University of Mississippi, University, MS 38677, USA; maelhend@olemiss.edu (M.A.E.); eibrahim@olemiss.edu (E.A.I.); aswanas@olemiss.edu (A.S.W.); aamarzou@olemiss.edu (A.A.M.); suman@olemiss.edu (S.C.); 2 Department of Agricultural Biochemistry, Faculty of Agriculture, Damietta University, Damietta 34726, Egypt; 3 Department of Biomolecular Sciences, School of Pharmacy, University of Mississippi, University, MS 38677, USA; 4 Pharmaceutical Analytical Chemistry Department, Suez Canal University, Ismailia 41522, Egypt; 5 Department of Pharmaceutical Chemistry, Faculty of Pharmacy, Al-Azhar University, Assiut Branch, Assiut 71524, Egypt; 6 Department of Chemistry and Biochemistry, University of Mississippi, University, MS 38677, USA; mgodfrey@olemiss.edu; 7 Department of Pharmaceutics and Drug Delivery, University of Mississippi, University, MS 38677, USA

**Keywords:** *Cannabis sativa*, flavonoids, cannflavins, HPLC-PDA, method validation

## Abstract

*Cannabis sativa* (*C. sativa*) is a psychoactive plant that has been used for millennia for medicinal, recreational, and industrial purposes. The main constituents of cannabis are the cannabinoids, with other constituents including terpenes and flavonoids that contribute to its bioactivity. Among the flavonoid class, there is a subclass, specific to cannabis, namely the cannflavins (A, B, and C), which are biologically active. This study is directed to the analysis of these constituents in various cannabis chemovars. In this study, an HPLC-PDA method was validated and applied to determine the content of cannflavins, namely, cannflavin A (CF-A), cannflavin B (CF-B), and cannflavin C (CF-C), in six different cannabis chemovars. The HPLC separation was achieved using a Luna^®^ C18 (150 × 4.6 mm × 3 μm) with isocratic elution using a mobile phase consisting of acetonitrile and water (65:35, *v*/*v*), both containing 0.1% formic acid at a flow rate of 1 mL/min, with the detector set at 342.4 nm. The method was validated according to the ICH guidelines and exhibited a linear relationship in the 5–500 ppm range with R^2^ > 0.99. The method showed good recovery, ranging from 82% to 98%. The intra-day and inter-day relative standard deviations (% RSDs) were ≤5.29%. Consequently, the method was applied for the determination of all these cannflavins in the different cannabis chemovars. CF-A was the most abundant cannflavin in the examined samples (15.2–478.38 ppm). The method was shown to be simple, accurate, and selective.

## 1. Introduction

*Cannabis sativa* (*C. sativa*) is a versatile medicinal plant that has been cultivated for medicinal, recreational, and industrial purposes since ancient times [1,2]. The plant has received great attention due to its medicinal applications as a therapeutic alternative for several health problems, such as chronic pain, as an analgesic, and as an appetite stimulant [3,4].

Cannabis has an extensive metabolic diversity from which more than 550 compounds have been identified [5]. Cannabinoids are the main secondary metabolites specific to cannabis and are responsible for the many medicinal values of the plant [6,7].

Δ^9^-tetrahydrocannabinol (∆^9^-THC) is the main psychoactive metabolite responsible for intoxication and has gained more interest as it is currently approved for use as an antiemetic for chemotherapy-related side effects (nausea and vomiting) and as an appetite stimulant in AIDS patients [8,9]. Cannabidiol (CBD) is the major non-psychoactive cannabinoid and, according to numerous studies, has been reported to have antioxidant, anti-inflammatory, anticonvulsant, and neuroprotective activities [10,11,12,13].

In addition to cannabinoids, a wide range of non-cannabinoid components have been identified in cannabis, including terpenes, flavonoids, spiroindans, stilbenoids, dihydrophenanthrenes, amino acids, sterols, and alkaloids [14,15].

More than thirty flavonoids have been isolated and identified from cannabis, primarily flavones and flavonols [14,16,17]. Cannflavins A, B, and C are examples of prenylated flavones that were isolated from cannabis [18,19]. Cannflavin A has also been identified in *Mimulus bigelovii*, a plant belonging to the Phrymaceae family [15,20]. Cannflavin B and 8-prenyl cannflavin B (dorsmanin D) were isolated from twigs of *Dorstenia mannii* [21].

Cannflavins A (CF-A), B (CF-B), and C (CF-C) are a class of prenylated flavonoids that accumulate mainly in the cannabis plant and have been reported to have anti-inflammatory [22] neuroprotective [23], and anti-proliferative activities, specifically, CF-A and CF-B [24,25]. CF-A demonstrated synergistic properties with other cannabinoids in the bladder cancer cell lines [26]. CF-A and CF-B are considered promising therapeutic agents in the treatment of Glioblastoma multiforme [27].

Preclinical studies have demonstrated that CF-A has anti-oxidative, neuroprotective, and antiviral activities, while CF-C exhibited antiparasitic activity [28,29,30].

**Figure 1:**
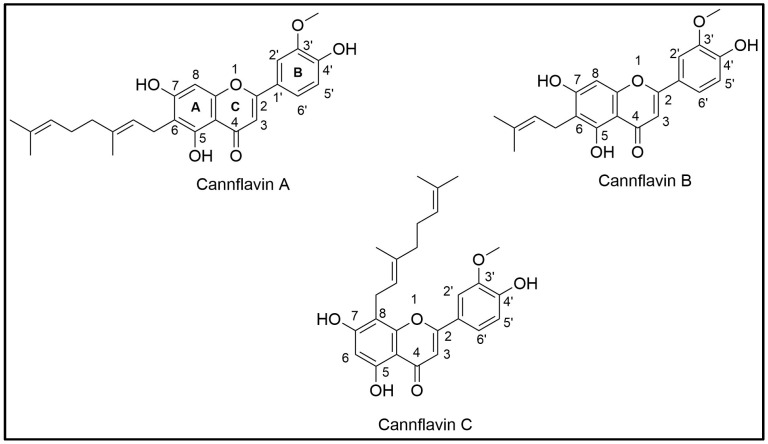
Chemical structures of cannflavins A, B, and C (CF-A, CF-B, and CF-C).

Flavonoids, including cannflavins, consist of C6–C3–C6 fundamental building rings, namely, rings A and B connected by a three-carbon ring, C (Figure 1) [31]. Cannflavins are biosynthesized from luteolin, which undergoes enzymatic methylation and prenylation [32,33].

CF-B and CF-C are exclusively found in cannabis [34,35,36], and CF-A is only found in one other plant besides cannabis, and their structure–activity relationship has shown distinct bioactivity attributed to the prenyl side chain (geranyl or prenyl moieties, respectively) at the C-6 [37], in addition to the methoxy group at the 3` position. Prenylation and methoxylation of the parent flavone increase the flavonoids’ lipophilicity and movement across the cell membrane, in addition to binding to receptors [16,36,38,39].

Qualitative and quantitative determination of cannflavins has been reported in numerous studies [40,41]. Ultra-high performance liquid chromatography (UHPLC) coupled with Q-Orbitrap high-resolution mass spectrometers (UHPLC-Q-Orbitrap HRMS) has been used for analysis of cannflavins in the polyphenolic fraction contained in polar extracts of four different commercial cultivars of hemp inflorescences [40]. Cannabinoids and cannflavins A and B have been analyzed using a targeted 1H NMR profiling method, along with two HPLC/PDA methods [41].

Moreover, the phenol composition of six hemp microgreens grown under a controlled environment, including CF-A and CF-B, has been determined using UHPLC-Q-Orbitrap HRMS [42].

Several analytical methods for cannflavin detection have been previously reported, including LC-DAD and LC-MS approaches by researchers such as Peschel, Croinin, and Pellati [41,43,44]. Croinin et al. utilized electrospray positive ionization in single-ion monitoring (SIM) mode, achieving excellent linearity and sensitivity within a short run time (<20 min). Their method was successfully applied to both hemp extracts and topical formulations, demonstrating practical utility across diverse matrices [44]. In contrast, our method builds upon this foundation by offering a broader calibration range (5–500 µg/mL vs. 0.5–2.5 µg/mL), enabling quantitation across a wider concentration spectrum. Moreover, the proposed method offers integration with HPLC-UV/PDA detection, which provides robust performance for routine analysis and complements mass spectrometric techniques. Additionally, application to complex cannabis matrices, with validated recovery and resolution of cannflavins alongside cannabinoids, has been carried out. These distinctions have been emphasized to clarify the novelty and analytical versatility of our approach.

Although Peschel et al.’s method provides valuable insight into the analytical challenges of cannflavin detection and quantification, the provided method has some limitations and challenges [41]. Unlike the moderate recovery rates reported by Peschel et al. (77.3–93.2%), which were considered suboptimal for cannflavin-focused analysis, our method achieved consistently higher recovery percentages ranging from 82% to 98%. This enhanced recovery supports the suitability of our assay for quantitative analysis of cannflavins, even within complex cannabis matrices. While Peschel et al. reported only CF-A and CF-B, our method successfully identified and quantified all three cannflavins, CF-A, CF-B, and CF-C. This expanded detection highlights the enhanced selectivity and resolution of our assay, particularly in complex cannabis matrices [41].

Pellati’s method showcases an impressive multi-compound approach, but because it spans cannabinoids, flavonoids, and terpenes, cannflavins were just one part of a broader profiling strategy. On the other hand, our method is tailored specifically for cannflavins A, B, and C, which allows for methodological advantages like higher selectivity and sensitivity for cannflavins, avoiding potential interference from other phytochemicals [43].

While MS-based methods offer greater sensitivity, not every analytical laboratory has access to mass spectrometry equipment [44]. Our HPLC-PDA method offers a practical and consistent approach for routine analysis, and the absence of CF-B and CF-C in certain plant parts probably indicates their low abundance compared to the method’s detection limits.

In light of this, the development and validation of reliable analytical methods are required for the understanding of the chemical profile of different cannabis chemovars and, consequently, the efficacy and safety for human use. The present study is aimed at the development and validation of a simple, accurate, and selective HPLC-UV/PDA and for the determination of the three cannflavins in different cannabis plant chemovars.

## 2. Experimental Design

### 2.1. Standards and Reagents

The three cannflavins reference standards, CF-A, CF-B, and CF-C, were isolated in-house at the National Center for Natural Products Research, University of Mississippi, from high-potency *C. sativa* chemovar plant material [15], and their chemical structures were confirmed as reported earlier by our group [15], as shown in Figure 1. The purity (≥99%) of the isolated cannflavins was confirmed using HPLC-PDA. All solvents (water, acetonitrile, acetone, hexanes, methanol, ethanol, and ethyl acetate) were HPLC grade and purchased from Fisher Scientific, Waltham, MA, USA.

### 2.2. Instrumentation

The HPLC-PDA system consisted of a Waters Alliance HPLC 2996 separation module (Waters Alliance, quaternary pump, vacuum degasser, and photodiode array detector), Waters^®^, Milford, MA, USA. The data were acquired and processed using Empower 3 software. The chromatographic separation was carried out using a Phenomenex Luna C18(2) (150 × 4.6 mm × 3 μm) analytical column. The mobile phase consisted of acetonitrile/water (65:35 *v*/*v*) containing 0.1% formic acid. The column temperature was maintained at 25 °C, the mobile phase flow rate was set at 1 mL/min, and the run time was 20 min. The injection volume was 10 μL, and all standards and samples were injected in triplicate. During each run, the PDA was set to acquire data from 190 to 400 nm, and the chromatograms were visualized at 342.4 nm.

The chosen wavelength was 342.4 nm, selected based on the maximum absorbance of the target analytes. Regarding baseline correction, the spectral baseline is internal to each sample injection, as processed by the Empower3 PDA software. This approach ensures consistent baseline handling across all injections without the need for external blank subtraction.

### 2.3. Preparation of Stock Standard Solutions

For the external standard calibration curve preparation, a stock standard solution of cannflavins mixture was prepared by weighing one mg of each standard in a 1 mL volumetric flask, dissolved in 500 µL of methanol, and completed to the mark with the same solvent to obtain a stock solution A (1 mg/mL).

### 2.4. Method Validation Procedure

For linearity and range, serial dilutions were made to prepare the external standard calibration points. The calibration curves were constructed using six calibration points (5–500 ppm) in triplicate. Based on the analysis of serial dilutions of a standard solution, the LOD and LOQ were determined to achieve S/N ratios of 3 and 10, respectively.

Accuracy and precision were evaluated by spiking standard cannflavins mixture into placebo cannabis plant materials (100 mg each) at three levels: low, medium, and high, corresponding to 100 ppm, 250 ppm, and 500 ppm, respectively. Placebo cannabis plant material refers to cannabis plant material (cannabis biomass free from cannabinoids, terpenes, flavonoids, and other constituents obtained by exhaustive solvent extraction with hexane followed by ethanol) that has been processed to remove or significantly reduce cannabis active metabolites while maintaining the physical appearance and matrix of the original plant.

An aliquot of each extract (10 µL) was injected and quantified using the prepared calibration curves. The intra-day and inter-day precisions were tested by injecting six replicates on the same day and for four consecutive days, and the relative standard deviation (%RSD) was calculated for each concentration level. Accuracy was calculated as % recovery at each concentration level on the same day, in addition to between days.

Method robustness was evaluated by observing the effects of the deliberate changes in method parameters, such as the flow rate ±0.2 mL/min and mobile phase composition (acetonitrile concentration by ±2 units) and the detection wavelengths (±3 nm).

### 2.5. Cannabis Plant Material

Six cannabis chemovars (high THC, high CBD, THC/CBD intermediate, high CBG, high THC/THCV, and high CBD/CBDV) were grown and harvested at the University of Mississippi (UM). The whole buds of the mature plants grown at UM were dried and manicured before analysis. Additionally, the high-CBG and high-CBD chemovars were analyzed at two distinct developmental stages: the vegetative stage and the flowering stage. During the vegetative stage, samples were collected from various plant parts, including leaves, stems, and roots. In the flowering stage, a more comprehensive analysis was conducted, with samples taken from leaves, stems, roots, buds, and pollen to assess the distribution of cannflavins across different tissues and developmental phases.

### 2.6. Sample Preparation for the Decarboxylation of Cannabis Plant Material

We considered sample pre-treatment necessary to reduce any interference from cannabinoid acids, especially CBDA, which was observed to elute with CF-A. Consequently, all cannabis plant material had to be decarboxylated to convert any amount of cannabinoid acids to neutral cannabinoids. The decarboxylation time was optimized for each chemovar to determine the optimum time at 130 °C for the decarboxylation process.

To perform the decarboxylation of 100 mg of each cannabis chemovar (high THC, high CBD, THC/CBD intermediate, and high CBG) in 9 replicates was weighed in a 20 mL scintillated glass vial, then, the vials were placed in an oven maintained at 130 °C for up to 80 min to determine the optimum experimental condition required to achieve complete decarboxylation. One vial was taken at 10 min time intervals starting from 0–80 min. Each vial was extracted using (3 × 2 mL of acetone) using an ultrasonic bath for 15 min. The supernatants were filtered using a 0.45-µm PFTE membrane filter. 10 µL was injected into the HPLC-PDA. The quantification of acid cannabinoids (CBDA, CBGA, and THCA) before and after decarboxylation was performed using the peak area ratio. The quantification was carried out using our previously validated method, employing UV–visible PDA detection, and 4-androstene-3,17-dione was used as the internal standard for quantitation of cannabinoid acids, based on peak area ratios [45]. Triplicate samples were analyzed for each reaction time using our previously published method [45].

### 2.7. Optimization of Cannflavin Extraction and Analysis

After the decarboxylation of all cannabis plant materials, 100 mg was extracted with different solvents, methanol, ethanol, ethyl acetate, and acetone (3 × 2 mL of each solvent), and sonicated for 15 min. Then the supernatants of each extraction solvent were combined and evaporated under a gentle stream of nitrogen. The dried residue after evaporation was reconstituted in 1 mL methanol, vortexed for 30 s, sonicated for 5 min at 30 °C, and filtered using a 0.45 μm PTFE membrane filter. The solution was transferred into an HPLC-vial and an aliquot (10 µL) was injected. The selection of the optimum solvent is based on the peak area of the extracted cannflavins.

### 2.8. Statistics

All tests were performed in triplicate. Data are expressed as mean ± standard deviation. Slopes and intercepts of calibration graphs were calculated by linear regression. Correlation coefficient (R^2^) values were obtained using the Microsoft Office Excel program. 2.1.

## 3. Results

### 3.1. Optimization of the HPLC-PDA Conditions

**Figure 2:**
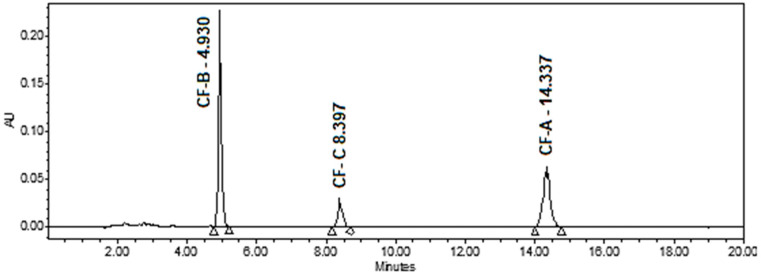
A representative HPLC chromatogram of a 75 μg/mL standard solution of cannflavins A, B, and C at λ 342.4 nm.

Different HPLC conditions were applied by selecting different columns, mobile phase compositions, column temperatures, and the PDA wavelengths. It was found that the Phenomenex Luna C18(2) (150 × 4.6 mm × 3 μm) column provided the best resolution and selectivity for the target cannflavins using a 65:35 acetonitrile/water mixture with 0.1% formic acid. Formic acid was added to the mobile phase (at 0.1%) to improve peak shape, reproducibility, and ionization efficiency. Specifically, it helps suppress silanol interactions on the column, which reduces peak tailing, especially for basic analytes. Additionally, it maintains a consistent acidic pH, contributing to stable retention times and better chromatographic reproducibility. The optimum column temperature was found to be 25 °C. A representative chromatogram of the cannflavin standard solution is shown in Figure 2.

**Figure 3:**
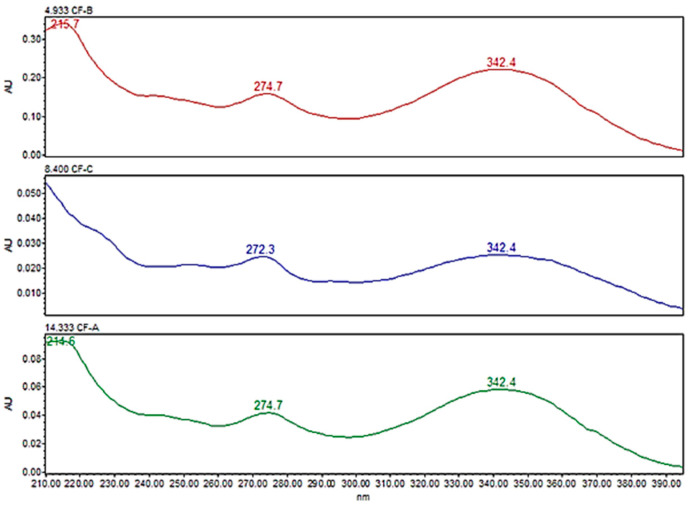
UV spectra (210–390 nm) for the cannflavins A, B, and C in acetonitrile/water 0.1% formic acid, using a PDA detector.

During the initial development of the method, the use of a PDA detector was beneficial for identifying lambda maxima (λmax) of cannflavins. The UV spectra of all the cannflavins exhibited three absorption maxima (λmax), at 220 nm, 274 nm, and 342.4 nm. The λmax, 342.4 nm, was selected as the wavelength for the quantification of cannflavins (Figure 3).

### 3.2. Optimization of Cannflavin Extraction

Different solvents, including methanol, ethanol, acetone, and ethyl acetate, were tested to determine the optimum solvent for cannflavins extraction.

**Figure 4:**
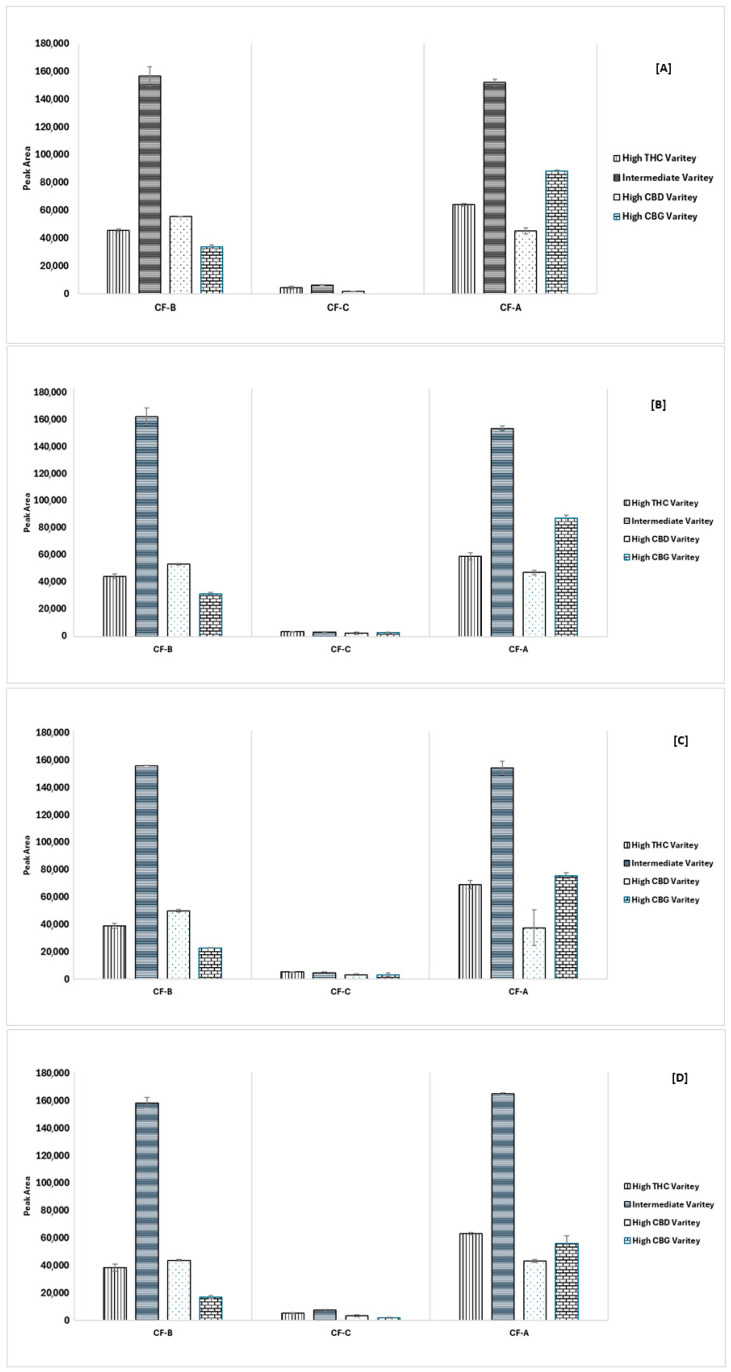
Extraction results of the target cannflavins using different solvents: (**A**) methanol extract, (**B**) ethanol extract, (**C**) acetone extract, and (**D**) ethyl acetate extract for the different chemovars.

These solvents, including ethyl acetate and acetone, show comparable performance for CF-B and CF-A in certain chemovars. Acetone consistently provided the highest overall recovery, particularly in the Intermediate Variety, as shown in Figure 4. The selection of acetone was based on its ability to extract all three target cannflavins with strong and reproducible peak areas, especially for CF-A, which showed poor recovery with other solvents. Additionally, acetone offered favorable chromatographic behavior and compatibility with the analytical method. However, ethyl acetate also performed well, but acetone was selected as the optimum solvent based on overall recovery and consistency across chemovars. The results of the extraction solvent optimization are shown in Figure 4. An extraction protocol has been adopted based on a previous study that demonstrated acetone as the best extraction solvent for cannflavin analysis.

### 3.3. Optimization of the Decarboxylation Process

We acknowledged the challenge in separating CBDA and CF-A due to their similar retention behavior under reversed-phase conditions. Multiple experimental adjustments were tested, including mobile phase composition and column temperature, but these did not yield sufficient resolution without compromising peak shape or sensitivity.

The coelution of CF-A and CBDA made it necessary to decarboxylate all the plant samples to convert all the CBDA into CBD, which elutes clearly from CF-A and thus, CF-A can be quantitatively determined without any interferences. The decarboxylation ensured full conversion of CBDA while maintaining the integrity of CF-A, thereby enhancing analytical specificity and reliability.

**Figure 5:**
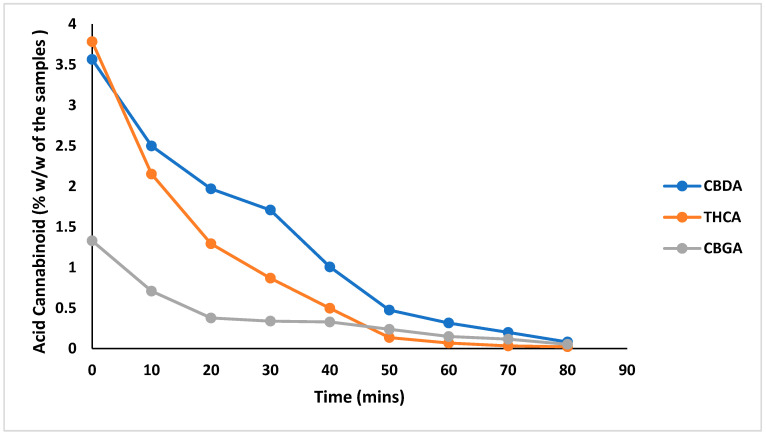
Decarboxylation profiles of acid cannabinoids: CBDA (in high CBD chemovar), THCA (in high THC chemovar), and CBGA (in high CBG chemovar), content expressed as % *w*/*w* of the sample over 90 min. heat treatment (CBDA; blue, THCA; orange, and CBGA; gray). All decarboxylation temperatures were carried out at 130 °C.

In the decarboxylation study, cannabis samples of the three chemovars (high CBD, high THC, and high CBG) were heated at different temperatures, and the cannabinoids were analyzed before and after decarboxylation. Complete decarboxylation of THCA was achieved at 70 min, while CBDA and CBGA were fully decarboxylated at 80 min. All decarboxylation temperatures were carried out at 130 °C (Figure 5).

It is worth noting that cannflavins, particularly CF-A and CF-B, are prenylated flavones that do not undergo decarboxylation like cannabinoid acids. However, they are thermally labile. As reported by Kuber and Jamuna (2023), cannflavin exposed to thermal stress showed insignificant instability (up to 3% degradation), indicating significant susceptibility to elevated temperatures [46]. In our study, heating was applied selectively to facilitate CBDA decarboxylation, and conditions were carefully optimized to minimize cannflavin degradation.

### 3.4. Method Validation Results

The method was validated following ICH guidelines Q2(R1) concerning linearity and range, LOD and LOQ, accuracy, precision, robustness, and selectivity [47].

#### 3.4.1. Linearity and Range

**Figure 6:**
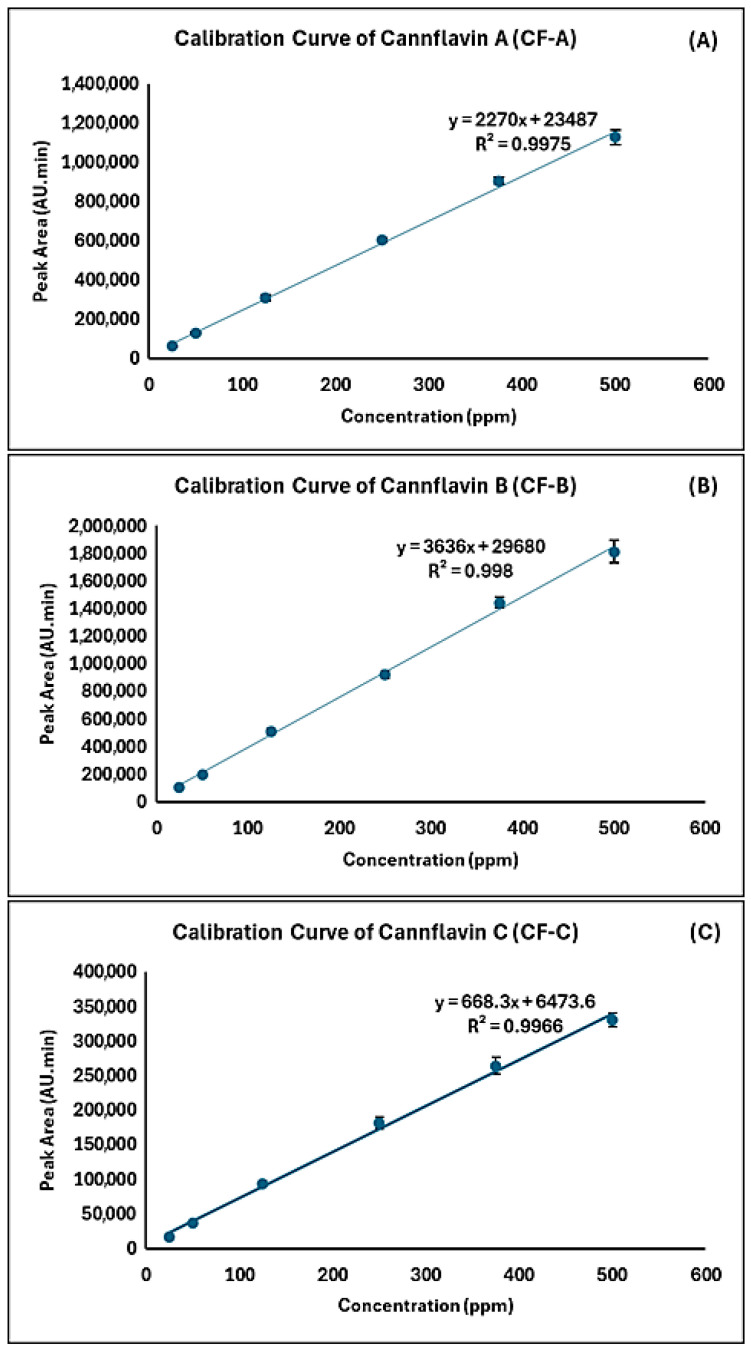
Calibration curves of CF-A, B, and C from 5 to 500 ppm are shown with standard deviation error bars (n = 3) for each concentration point. (**A**) calibration curve of CF-A; (**B**) calibration curve of CF-B; (**C**) calibration curve of CF-C.

External calibration curves were constructed by plotting the peak area of each cannflavin versus the concentration in triplicate. All the analyzed cannflavins standards were linear over the dynamic concentration range of 5–500 ppm with correlation coefficients (R^2^) of ≥0.997 (Table 1). The calibration curves are displayed in Figure 6.

#### 3.4.2. LOD and LOQ

HPLC-PDA Sensitivity: The photodiode array detector typically provides detection limits (LOD) in the low microgram per milliliter (µg/mL) range, depending on the compound’s chromophoric properties and matrix complexity. In our study, the LODs for CF-A, CF-B, and CF-C were approximately 1.5, 1.2, and 1 ppm, respectively, which restricted their detectability in certain plant tissues. Conversely, MS-based methods, especially LC-MS/MS, can reach LODs in the nanogram per milliliter range or lower, offering much higher sensitivity. For example, previous studies have reported LODs for flavonoids and cannabinoids around 500 ng/mL using a single quadrupole mass spectrometer [44], and an ion trap mass analyzer with an ESI ion source, where the LOQ of cannflavins was 1.3 µg/mL [43].

The method showed good sensitivity as the LOD and LOQ ranged from 1–1.5 ppm and 3.3–5 ppm, respectively, using the respective S/N ratios of 3 and 10. The results of LOD and LOQ were shown in Table 1.

#### 3.4.3. Accuracy and Precision

Accuracy results have shown an acceptable % recovery, which ranged from 82–98% (Table 2 and Table 3). The method showed good precision as the intra-day precisions ranged from 0.23–5.29%, while the inter-day precision ranged from 1.14–3.34%.

#### 3.4.4. Selectivity

**Figure 7:**
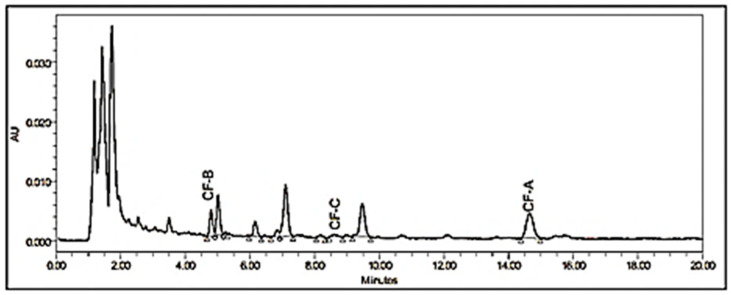
HPLC-PDA chromatogram of cannabis extract of high THC chemovar at 342.4 nm based on the maximum absorbance of the target analytes. The spectral base is internal to each sample injection, as processed by the PDA software.

Selectivity of the LC-PDA method was evaluated following ICH Q2(R1) guidelines through chromatographic resolution and UV spectral comparisons. Specifically, selectivity was confirmed by demonstrating that cannflavins A, B, and C produced well-resolved peaks at distinct retention times (CF-B: 4.93 min, CF-C: 8.39 min, CF-A: 14.33 min), with resolution factors >1.5 between adjacent peaks (Figure 3 and Figure 7). The method displayed selectivity as it can separate all the targeted analytes from each other and the plant matrix, as illustrated in Figure 7. Furthermore, the full spectrum of each cannflavin in the samples was identical to that of the standard, indicating no other overlapping peaks. Representative chromatograms of high THCV, high CBDV, high CBD, and high CBG are shown in Figures S1, S2, S3, S4, and S5 respectively.

The chosen wavelength for the chromatogram in Figure 7 was 342.4 nm, selected based on the maximum absorbance of the target analytes (as depicted in Figure 3). Regarding baseline correction, the spectral baseline is internal to each sample injection, as processed by the PDA software. This approach ensures consistent baseline handling across all injections without the need for external blank subtraction.

#### 3.4.5. Method Robustness

Small changes in method parameters such as the flow rate, mobile phase composition, and the detection wavelength had no effects on the measured analyte responses in terms of peak area.

Chromatographic response is highly sensitive to several experimental parameters, each of which can significantly affect retention time, peak area, and resolution. In our study, we carefully optimized the following conditions.

- Mobile Phase Composition: The polarity and strength of the mobile phase directly affect analyte interaction with the stationary phase. A higher organic content speeds up elution, reducing retention time but may lower resolution, especially for CF-B and CF-C. Conversely, a lower organic content enhances separation but extends the run time. We chose a mobile phase of acetonitrile and water (65:35, *v*/*v*), both containing 0.1% formic acid, at a flow rate of 1 mL/min, with the detector set at 342.4 nm to balance these effects and ensure baseline resolution of CF-B, CF-C, and CF-A.
- Flow Rate: Increasing the flow rate shortens retention times and can decrease peak width, but it may also cause coelution or loss of resolution. Lower flow rates improve separation but increase analysis time. Our flow rate was optimized to maintain peak symmetry and reproducibility.
- Column Temperature: Although our method was performed under ambient conditions, temperature can influence analyte volatility and interaction with the stationary phase. Elevated temperatures typically reduce retention times and may affect peak shape, especially for thermally sensitive compounds like cannflavins.
- Injection Volume and Solvent Strength: Large injection volumes or strong solvents can distort peak shapes and reduce efficiency. We used minimal injection volumes and matched the solvent strength to the initial mobile phase to preserve chromatographic integrity.
- Stationary Phase Selection: The choice of column chemistry (e.g., C18 reversed phase) determines selectivity and retention behavior. CF-A and CBDA exhibited similar retention under our conditions, necessitating decarboxylation to resolve the coelution.

### 3.5. Analysis of Cannflavins in Six Cannabis Chemovars

The validated method was extended to the analysis of cannflavins A, B, and C in different cannabis plant materials, and the concentrations of cannflavins in the different cannabis chemovars (high CBD chemovar, THC/CBD intermediate chemovar, high THC chemovar, and high CBG chemovar) were determined.

The validated HPLC-PDA method was employed to quantify cannflavins A (CF-A), B (CF-B), and C (CF-C) across 22 cannabis chemovar samples representing diverse chemovars, including high-CBD, THC/CBD intermediate, high-THC, high-CBG, THC/THCV, and CBD/CBDV chemovars. Acceptable results were obtained in plant matrices as proven by the % recoveries (Table 2 and Table 3). Moreover, the chromatograms showed no interference in the form of coeluted peaks at the retention time of the target analytes (Figure 7). The results (Table 4) demonstrate substantial variability in cannflavin profiles, both within and between chemovars, reflecting chemovars’ differences in secondary metabolite accumulation.

In high-CBD chemovar samples, CF-A was the predominant flavonoid detected, ranging from 37.48 ppm in HCBD3 to 60.10 ppm in HCBD1 (Table 4). CF-B levels were more consistent, ranging from 14.34 to 17.24 ppm, while CF-C content varied widely, from 7.38 ppm in HCBD5 to 29.62 ppm in HCBD3 (Table 4). The data suggest that while CF-A is relatively abundant across all five samples, CF-C levels may be more chemovar-specific, indicating metabolic pathway differences or gene expression variability influencing CF-C biosynthesis.

Samples within the THC/CBD intermediate chemovar displayed the highest CF-A among all chemovar categories. CF-A ranged widely from 21.42 ppm (IM4) to 387.44 ppm (IM3), with IM3 showing the highest level of all measured flavonoids in the entire dataset. CF-B and CF-C were also elevated in these samples, reaching 140.82 ppm and 61.85 ppm, respectively, in IM3 (Table 4).

In high-THC chemovar samples, CF-A concentrations varied between 38.87 and 162.63 ppm, with HTHC5 showing the highest CF-A concentration within this group. CF-B ranged from 11.20 to 53.40 ppm, and CF-C from 12.45 to 28.58 ppm (Table 4).

High-CBG Chemovar, CF-A concentrations ranged from 15.89 ppm in HCBG1 to 148.34 ppm in HCBG2 (Table 4). Interestingly, CF-B was below the limit of quantification (bLOQ) in HCBG1 and below the limit of detection (bLOD) in HCBG3 to HCBG5, indicating that some high-CBG chemovars may be deficient in CF-B production. CF-C was also undetectable (bLOD) in three of the five samples, reinforcing the idea that high-CBG chemotypes may selectively favor CF-A synthesis over other flavonoids.

The THC/THCV chemovar (HTHCV1) showed elevated levels of CF-A (150.54 ppm) and moderate amounts of CF-B (38.38 ppm) and CF-C (15.88 ppm), positioning it closer to the high-THC profile in terms of flavonoid composition. Similarly, the CBD/CBDV chemovar (HCBDV1) had one of the highest CF-A levels detected (181.68 ppm), with CF-B at 101.55 ppm and CF-C at 9.60 ppm (Table 4). Overall, CF-A was consistently the most abundant cannflavin across all chemovars, with levels ranging from 15.89 ppm to 387.44 ppm. In contrast, CF-B and CF-C exhibited more chemovar-dependent patterns, with some samples lacking detectable levels. The distinct distribution of these flavonoids across chemotypes highlights the metabolic diversity within *C. sativa* and underscores the potential for targeted breeding or selection strategies to enhance specific flavonoid profiles.

Overall, the variability of CF compounds (CF-A, CF-B, CF-C) across cannabis chemovars demonstrates distinct biosynthetic patterns that may affect both therapeutic potential and product safety. For example, CF-A levels vary significantly—from as low as 15.89 μg/g in HCBG1 to as high as 387.44 μg/g in IM3—highlighting chemovar-specific expression. Similarly, CF-B and CF-C amounts show notable fluctuations, with CF-B reaching 140.82 μg/g in IM3 and CF-C peaking at 61.85 μg/g in the same chemovar, while remaining below the limit of detection in others, such as HCBG3–HCBG5. These differences suggest that some chemovars may offer enriched profiles of minor cannabinoids, potentially contributing to unique pharmacological effects or influencing the activity of major cannabinoids. From a safety standpoint, while CF compounds are not known to be overtly toxic, their concentration in high-potency extracts could affect tolerability or interact with other constituents. Therefore, profiling CF variability is crucial for chemovar selection in product development, ensuring consistency, and predicting therapeutic or adverse outcomes.

### 3.6. Analysis of Cannflavins in Samples from Different Parts of the Female Cannabis Plant

Samples from different parts of female cannabis plants (leaves, stems, roots, and buds), as well as pollen grains from male plants, were analyzed to detect seasonal changes in cannflavin content. The analysis results for high CBG and high CBD chemovars throughout the growing season, including both vegetative and flowering stages, are presented in Table 5.

During the vegetative stage, leaves from high CBG and high CBD chemovars contained the highest levels of their characteristic cannflavins, particularly CF-A, with concentrations of 133.76 ppm and 27.79 ppm, respectively (Table 5). CF-B concentrations were higher in the high CBG chemovar samples (27.52 ppm) compared to the high CBD chemovar (3.84 ppm). CF-C was detected in relatively small amounts (10.26 ppm) only in the high CBD chemovar. In contrast, buds from the high CBG chemovar had higher amounts of the tested cannflavins, with no CF-C detected (Table 5).

In the flowering stage, cannflavin concentrations decreased in the leaves and increased in the buds for both chemovars. In the high CBG chemovar samples, only CF-A (51.82 ppm) and CF-B (4.99 ppm) were detected in the leaves (Table 5). In the buds of high CBG chemovar samples, CF-A showed the highest concentration at 478.38 ppm, followed by CF-B at 75.39 ppm and CF-C at 49.34 ppm (Table 5). In the high CBD chemovar samples, CF-A was present in the leaves at 15.20 ppm, while both CF-B and CF-C were below the limit of detection. In the buds, CF-A and CF-B were measured at 18.47 ppm and 26.63 ppm, respectively, while CF-C remained below the detection limit (Table 5).

A comparative evaluation of the cannflavin concentrations across different cannabis chemovars in our study versus the published literature data showed substantial variability in levels linked to plant genotype, tissue type, and analytical approach. In the present study dataset, CF-A concentrations ranged from 15.89 ppm (HCBG1) to 387.44 ppm (IM3), while CF-B ranged from 11.20 to 140.82 ppm, and CF-C ranged from 3.39 to 61.85 ppm. Notably, intermediate chemovars (e.g., IM3 and IM2) and CBDV-rich chemovars (e.g., HCBDV1) exhibited the highest levels of CF-A and CF-B, consistent with enhanced flavone accumulation in inflorescences. These findings align closely with the results reported by Pellati et al. [43], where CF-A levels were 59.9–318.9 ppm and CF-B levels of 25.3–412.2 ppm in hemp samples of different varieties. Further, our data were similar to those of Luana et al., who found mean values of 61.8 ppm (CF-A) and 84.5 ppm (CF-B) in commercial cannabis inflorescences using UHPLC-Q-Orbitrap HRMS [40]. In contrast, Pollastro et al. [48] documented significantly lower concentrations in leaf tissues, with only 6 ppm of CF-A and 0.8 ppm of CF-B, reinforcing the view that inflorescences are the primary reservoir of flavones in the cannabis plant. Moreover, a study by Conor et al. also analyzed hemp extracts for CF-A, CF-B, and CF-C and found levels ranging from 93.55 to 197.03 parts per million, CF-B between 7.19 and 46.21 parts per million, and CF-C between 0.57 and 1.36 parts per million. However, CF-C levels reported by Conor et al. were significantly lower, suggesting significant variations in CF-C accumulation across hemp varieties [44].

Overall, the data suggest that cannflavins’ content is highly chemovar-dependent, with intermediate and CBD/CBDV chemovar showing the greatest potential for cannflavins’ accumulation (CF-A and CF-B), and that inflorescences contain substantially higher levels than other tissues such as leaves.

Pollen grains from mature male plants during the flowering stage contained all tested cannflavins, with CF-A, CF-B, and CF-C concentrations at 117.95 ppm, 14.98 ppm, and 12.85 ppm, respectively (Table 5). Cannflavins in both stems and roots were below the limit of detection.

## 4. Conclusions

A simple, accurate, precise, rapid, and reproducible HPLC-PDA method was developed and validated for the identification and quantitative analysis of cannflavins A, B, and C in different cannabis chemovars and at different growing stages. The method selectivity was evident in its ability to determine the levels of cannflavins in the presence of other complex components present in the cannabis plant material, with peak purity determined from full UV spectra of each cannflavin. It was found that cannflavins are mainly (if not exclusively) found in the leaves and buds of the cannabis plant as well as in the pollen of the male plant, with cannflavin A being the most predominant in all cannabis chemovars.

## Figures and Tables

**Table 1:** Retention time (min.), regression equation parameters, linearity range, correlation coefficient (R2), LOD, and LOQ.

No	Cannflavins	R_t_ (min.)	Regression Equation	R^2^	Linearity Range (ppm)	LOD (ppm)	LOQ (ppm)
**1**	CF-A	14.337	y = 2270x + 23,487	0.9975	5–500	1.5	5.0
**2**	CF-B	4.930	y = 3636x + 29,680	0.9980	5–500	1.2	4.0
**3**	CF-C	8.397	y = 668.3x + 6473.6	0.9966	5–500	1.0	3.3

**Table 2:** Intra-day accuracy and precision for cannflavins following the assay method.

Time	Day 1			Day 2			Day 3			Day 4		
Cannflavins	CF-A	CF-B	CF-C	CF-A	CF-B	CF-C	CF-A	CF-B	CF-C	CF-A	CF-B	CF-C
	487.20	499.00	473.80	488.20	490.70	465.90	485.40	491.30	472.00	480.90	481.80	465.00
500 ppm	484.70	496.70	481.80	482.70	495.60	480.10	478.10	487.10	467.50	473.00	477.30	451.40
	482.70	487.60	479.10	491.10	492.20	486.10	477.70	488.80	463.30	470.60	476.80	459.40
Average	484.87	494.43	478.23	487.33	492.83	477.37	480.40	489.07	467.60	474.83	478.63	458.60
SD	2.25	6.03	4.07	4.27	2.51	10.37	4.33	2.11	4.35	5.39	2.75	6.84
% RSD	0.46	1.22	0.85	0.88	0.51	2.17	0.90	0.43	0.93	1.13	0.58	1.49
% Recovery	96.97	98.89	95.65	97.47	98.57	95.47	96.08	97.81	93.52	94.97	95.73	91.72
	214.80	224.70	210.00	220.60	225.00	214.40	216.30	219.80	213.60	215.10	217.80	201.90
250 ppm	218.30	223.10	214.80	219.60	224.70	218.90	213.20	219.50	216.70	213.90	216.80	210.40
	217.60	223.40	216.70	219.50	223.40	216.90	208.60	216.60	209.20	215.80	217.20	210.40
Average	216.90	223.73	213.83	219.90	224.37	216.73	212.70	218.63	213.17	214.93	217.27	207.57
SD	1.85	0.85	3.45	0.61	0.85	2.25	3.87	1.77	3.77	0.96	0.50	4.91
% RSD	0.85	0.38	1.61	0.28	0.38	1.04	1.82	0.81	1.77	0.45	0.23	2.36
% Recovery	86.76	89.49	85.53	87.96	89.75	86.69	85.08	87.45	85.27	85.97	86.91	83.03
100 ppm	90.50	92.70	91.40	86.80	89.70	89.90	84.00	90.00	88.60	83.60	89.20	92.10
	89.50	93.20	95.40	85.40	90.40	87.90	84.10	87.50	80.50	83.40	89.40	83.80
	87.70	92.10	90.00	82.60	89.60	89.00	81.90	87.50	87.10	82.40	87.80	84.20
Average	89.23	92.67	92.27	84.93	89.90	88.93	83.33	88.33	85.40	83.13	88.80	86.70
SD	1.42	0.55	2.80	2.14	0.44	1.00	1.24	1.44	4.31	0.64	0.87	4.68
% RSD	1.59	0.59	3.04	2.52	0.48	1.13	1.49	1.63	5.05	0.77	0.98	5.40
% Recovery	89.23	92.67	92.27	84.93	89.90	88.93	83.33	88.33	85.40	83.13	88.80	86.70

**Table 3:** Inter-day accuracy and precision for cannflavins following the HPLC method.

Level	High Concentration				Medium Concentration				Low Concentration			
	500 ppm				250 ppm				100 ppm			
**Cannflavins**	Average	SD	% RSD	% Recovery	Average	SD	% RSD	% Recovery	Average	SD	% RSD	% Recovery
**CF-A**	481.86	3.23	0.67	96.37%	216.11	2.68	1.24	86.44%	85.16	3.19	3.74	85.16%
**CF-B**	488.74	7.10	1.45	97.75%	221.00	3.58	1.62	88.40%	89.93	1.94	2.16	89.93%
**CF-C**	470.45	8.62	1.83	94.09%	212.83	4.76	2.24	85.13%	88.33	2.72	3.08	88.33%

SD = standard deviation; % RSD = relative standard deviation.

**Table 4:** Analysis results of cannflavin content (ppm) in *C. sativa* chemovars.

Chemovar		CF-A		CF-B		CF-C	
		Mean	SD	Mean	SD	Mean	SD
**High CBD chemovar**	HCBD1	60.10	0.65	17.24	0.23	16.39	0.86
	HCBD2	49.52	1.05	15.79	0.28	13.17	0.95
	HCBD3	37.48	1.05	14.87	0.18	29.62	0.46
	HCBD4	54.15	3.56	16.05	0.01	18.34	3.59
	HCBD5	55.82	0.41	14.34	0.19	7.38	0.59
**THC/CBD Intermediate chemovar**	IM1	58.45	0.70	38.95	0.11	9.60	0.77
	IM2	141.78	1.93	66.71	4.21	18.51	2.27
	IM3	387.44	0.65	140.82	1.21	61.85	0.54
	IM4	21.42	0.42	11.46	0.26	5.29	0.89
	IM5	99.47	2.91	55.47	0.38	12.41	1.07
**High THC chemovar**	HTHC1	142.42	0.92	40.74	0.07	25.70	2.61
	HTHC2	38.87	0.85	11.20	0.07	12.45	1.85
	HTHC3	128.62	2.06	53.40	1.98	25.11	1.29
	HTHC4	94.76	2.38	35.21	2.00	17.75	0.02
	HTHC5	162.63	1.09	50.15	3.41	28.58	1.05
**High CBG chemovar**	HCBG1	15.89	0.45	bLOQ	bLOD	3.39	0.04
	HCBG2	148.34	0.78	17.40	0.75	13.95	1.10
	HCBG3	88.48	0.50	13.69	0.81	bLOD	bLOD
	HCBG4	92.50	0.51	11.89	0.72	bLOD	bLOD
	HCBG5	108.05	1.52	12.85	0.21	bLOD	bLOD
**THC/THCV chemovar**	HTHCV1	150.54	3.11	38.38	0.11	15.88	0.19
**CBD/CBDV chemovar**	HCBDV1	181.68	2.07	101.55	1.21	9.60	0.06

bLOD = below limit of detection; bLOQ = below limit of quantification.

**Table 5:** Cannflavins’ content (ppm) of different parts of female and male plants of different cannabis chemovars, in addition to pollen grains from the male plant.

Vegetative Stage				Flowering Stage			
Chemovar	High CBG Chemovar						
**Plant Part**	**CF-A**	**CF-B**	**CF-C**	**Plant Part**	**CF-A**	**CF-B**	**CF-C**
Leaves	133.76	27.52	10.26	Leaves	51.82	4.99	bLOD
Stem	bLOD	bLOD	bLOD	Stem	bLOD	bLOD	bLOD
Roots	bLOD	bLOD	bLOD	Roots	bLOD	bLOD	bLOD
				Buds	478.38	75.39	49.34
**Chemovar**	**High CBD Chemovar**						
**Plant Part**	**CF-A**	**CF-B**	**CF-C**	**Plant Part**	**CF-A**	**CF-B**	**CF-C**
Leaves	27.79	3.84	bLOD	Leaves	15.20	bLOD	bLOD
Stem	bLOD	bLOD	bLOD	Stem	bLOD	bLOD	bLOD
Roots	bLOD	bLOD	bLOD	Roots	bLOD	bLOD	bLOD
				Buds	18.47	28.63	bLOD
				**Male Plant**			
				**Plant Part**	**CF-A**	**CF-B**	**CF-C**
				Leaves	bLOD	5.95	bLOD
				Stem	bLOD	bLOD	bLOD
				Roots	bLOD	bLOD	bLOD
				pollen	117.95	14.98	12.85

bLOD = below limit of detection.

## Data Availability

Data are contained within the article.

## Supplementary Materials

The following supporting information can be downloaded at: https://www.mdpi.com/article/10.3390/mps8050100/s1, Figure S1: HPLC-PDA chromatogram of cannabis extract of high THCV chemovar at 342.4 nm; Figure S2: HPLC-PDA chromatogram of cannabis extract of high CBDV chemovar at 342.4 nm; Figure S3: HPLC-PDA chromatogram of cannabis extract of high CBD chemovar at 342.4 nm; Figure S4: HPLC-PDA chromatogram of cannabis extract of high CBG chemovar at 342.4 nm; Figure S5: HPLC-PDA chromatogram of cannabis extract of intermediate chemovar at 342.4 nm.

## Abbreviations

The following abbreviations are used in this manuscript:

∆^9^-THC	Δ^9^-tetrahydrocannabinol
bLOD	Below the Limit of Detection
bLOQ	Below the Limit of Quantification
*C. sativa*	*Cannabis sativa*
CBD	Cannabidiol
CBDA	Cannabidiolic Acid
CBG	Cannabigerol
CBGA	Cannabigerolic Acid
CF-A	Cannflavin-A
CF-B	Cannflavin-B
CF-C	Cannflavin-C
HCBD	High CBD Chemovar
HCBG	High CBG Chemovar
HPLC-PDA	High Performance Liquid Chromatography–Photodiode Array Detector
HTHCV	High THC/THCV Chemovar
IM	High THC/CBD Intermediate Chemovar
LOD	Limit of Detection
LOQ	Limit of Quantification
ppm	Parts Per Million
RSD	Relative Standard Deviation
SD	Standard Deviation
THC	Tetrahydrocannabinol
THCA	Tetrahydrocannabinolic Acid
THCV	Tetrahydrocannabivarin
