# Supplementary material for: Development and Validation of an HPLC–UV/PDA Method for the Determination of Cannflavins in Different Cannabis sativa Chemovars
Source: Methods Protoc. 2025 Sep 3;8(5):100. doi: 10.3390/mps8050100 (PMC12452342; doi:10.3390/mps8050100)
Supplement: Supplementary file 1 [file mps-08-00100-s001.zip › mps-3763513-supplementary.pdf]

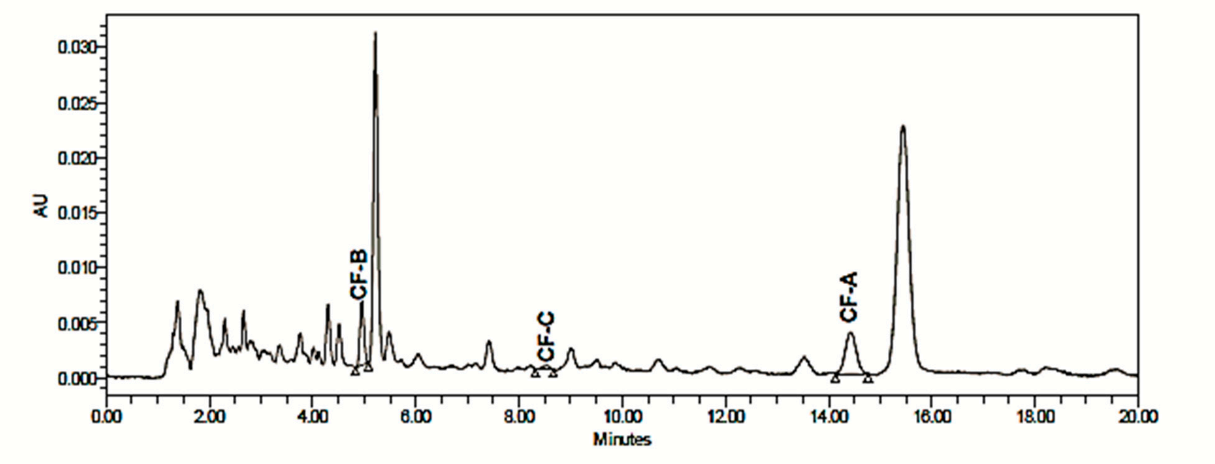

Figure S1. HPLC-PDA chromatogram of cannabis extract of high THCV chemovar at 342.4 nm

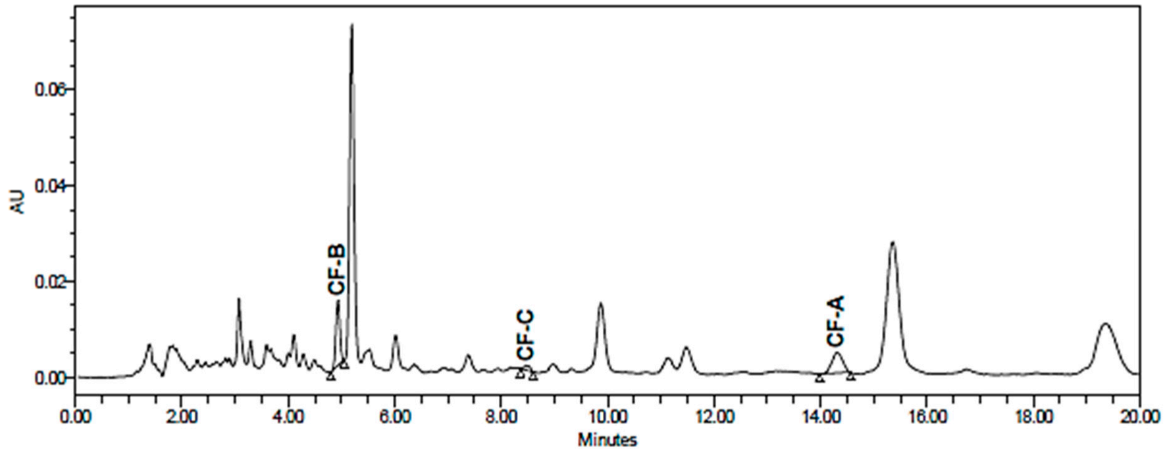

Figure S2. HPLC-PDA chromatogram of cannabis extract of high CBDV chemovar at 342.4 nm

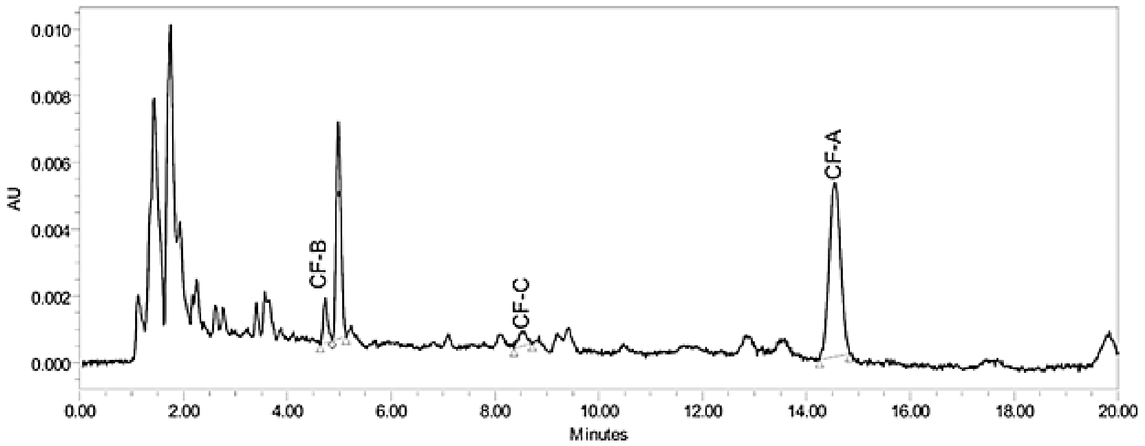

Figure S3. HPLC-PDA chromatogram of cannabis extract of high CBD chemovar at 342.4 nm

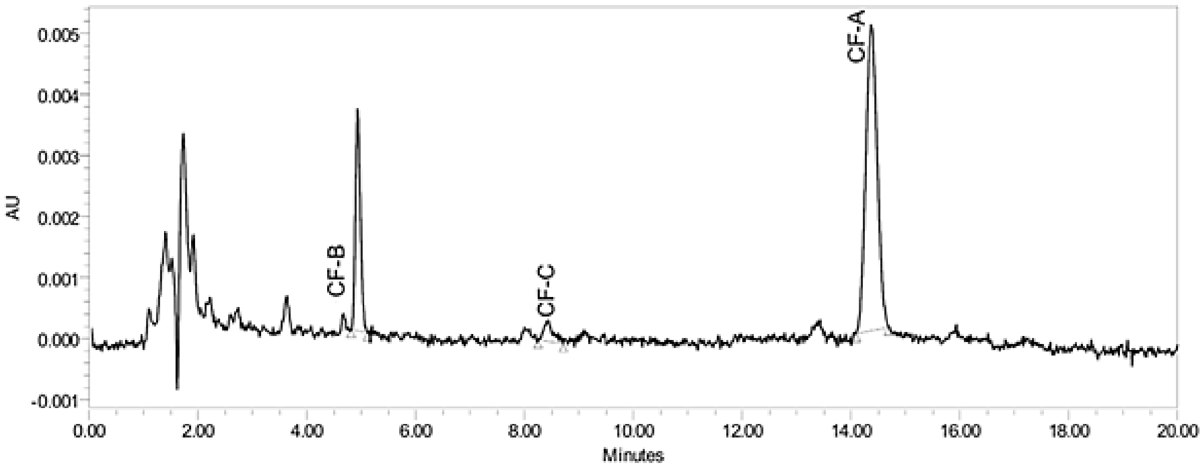

Figure S4. HPLC-PDA chromatogram of cannabis extract of high CBG chemovar at 342.4 nm

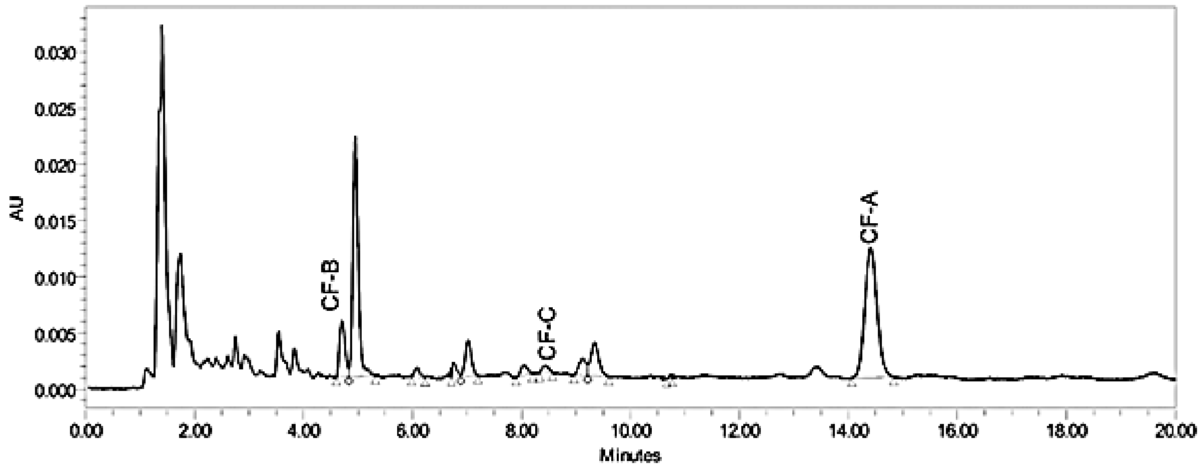

Figure S5. HPLC-PDA chromatogram of cannabis extract of intermediate chemovar at 342.4 nm
